# Complete Chloroplast Genome Characterization of *Oxalis Corniculata* and Its Comparison with Related Species from Family Oxalidaceae

**DOI:** 10.3390/plants9080928

**Published:** 2020-07-23

**Authors:** Lubna, Sajjad Asaf, Rahmatullah Jan, Abdul Latif Khan, In-Jung Lee

**Affiliations:** 1 Department of Botany, Garden Campus, Abdul Wali Khan University, Mardan 23200, Pakistan; lubnabilal68@gmail.com; 2 Natural and Medical Sciences Research Center, University of Nizwa, Nizwa 616, Oman; 3 School of Applied Biosciences, Kyungpook National University, Daegu 41566, Korea; rehmatbot@yahoo.com

**Keywords:** Oxalidaceae, chloroplast genome comparison, inverted repeats, divergence, SSRs, phylogeny

## Abstract

*Oxalis corniculata* L. (family Oxalidaceae) is a small creeper wood sorrel plant that grows well in moist climates. Despite being medicinally important, little is known about the genomics of this species. Here, we determined the complete chloroplast genome sequence of *O. corniculata* for the first time and compared it with other members of family Oxalidaceae. The genome was 152,189 bp in size and comprised of a pair of 25,387 bp inverted repeats (IR) that separated a large 83,427 bp single copy region (LSC) and a small 16,990 bp single copy region (SSC). The chloroplast genome of *O. corniculata* contains 131 genes with 83 protein coding genes, 40 tRNA genes, and 8 rRNA genes. The analysis revealed 46 microsatellites, of which 6 were present in coding sequences (CDS) regions, 34 in the LSC, 8 in the SSC, and 2 in the single IR region. Twelve palindromic repeats, 30 forward repeats, and 32 tandem repeats were also detected. Chloroplast genome comparisons revealed an overall high degree of sequence similarity between *O. corniculata* and O. drummondii and some divergence in the intergenic spacers of related species in Oxalidaceae. Furthermore, the seven most divergent genes (*ccs*A, *clp*P, *rps*8, *rps*15, *rpl*22, *mat*K, and *ycf*1) among genomes were observed. Phylogenomic characterization on the basis of 60 shared genes revealed that *O. corniculata* is closely related to *O. drummondii*. The complete *O. corniculata* genome sequenced in the present study is a valuable resource for investigating the population and evolutionary genetics of family Oxalidaceae and can be used to identify related species.

## 1. Introduction

The largest genus of family Oxalidaceae is *Oxalis* L., which is distributed mostly in Southern Africa and South America, and comprises of more than 500 species. About one-half of the total species (>200 spp) growing in Southern Africa share a bulbous or tuberous in herbaceous taxa [1,2,3]. A huge morphological variation have been observed among approximately 250 species in South America, where this genus seems to have originated and diversified [2,4]. However, *Oxalis* sections Corniculatae DC. consist of creeping herbs, and many of the species grows in the temperate and humid areas of the Americas [5]. A cytogenetic study showed that Corniculatae tends to be categorized into two sub-groups: (1) a large number of diploid and polyploid species with a base chromosome number of *x* = 6, symmetrical karyotypes, and medium and small chromosomes size, and (2) a minor number of diploid species having *x* = 5, more asymmetrical karyotypes, and average to large chromosomes [3,6,7]. The taxonomy has been suffering with similarities in phenotypes across different species.

Among species from genus *Oxalis*, *Oxalis corniculata* L. is a small creeping herb that is adaptive to moist conditions with yellow flowers and cylindrical fruits [4]. The origin of *O. corniculata* L. has been unknown until now. *O. corniculata* was first described by Carl Linnaeus from the Mediterranean region and various others suggested that this region is the native range [4,8]. Currently, among vascular plant species, *O. corniculata* has the third largest distribution [9]. The early spread and global distribution of *O. corniculata* has contributed to the difficulty in identifying its native range [4]. It is highly persistent in the horticulture industry and commonly found in gardens and as a hitchhiker in plant pots in gardens and nurseries [10]. Additionally, *O. corniculata* had medicinal values to treat various infectious diseases [11]. It has been known as antifungal, antibacterial, anti-inflammatory, anthelmintic, antidiuretic, astringent, emmenagogue, depurative, febrifuge, lithontriptic, stomachic, and styptic [12]. It has sticky seeds, explosive capsules, persistent flowers, and a brief life cycle, which make it an effective colonizer and constant weed. *O. corniculata* is not a strong competitor; however, its abundance and enormous range increase its overall impact as a weed.

Chloroplasts (cp) are important plant organelles that carry out photosynthesis and the biosynthesis of amino acid, fatty acids, and pigments [13,14]. Similarly, cp genomes have proven to be a valuable resource for species identification, plant phylogenetics, population genetics, and genetic engineering. Its DNA is inherited maternally in not all but in the majority of angiosperm species [15]. As a result of its inheritance approach, cp DNA plays a significant role in population genetics and molecular evolution. Therefore, its DNA can not only be used for species discrimination but can also be used solved many questions related to taxonomy [15,16]. Chloroplasts contain their own independent genomes and genetic systems, and DNA replication and transmission to daughter organelles result in a cytoplasmic inheritance of characteristics associated with primary events in photosynthesis [17,18]. The cp genome structure of angiosperms is circular—about 120–217 kb in size—and contain a quadripartite conformation [19,20]. It has small single copy (SSC) and large single copy (LSC) regions that are generally segregated by double copies of an inverted repeat region (IRa and IRb) [20]. In terms of gene order and content, the cp genome is usually conserved in various families of the angiosperm, such as Campanulaceae, Fabaceae, Geraniaceae, and Oleaceae [21,22,23]. Due to its conserved structure, small size, and recombination-free nature, the chloroplast genome is broadly used in plant phylogenetic analysis [24,25]. Similarly, the cp genome has a highly conserved structure that simplifies sequencing and primer designing, and chloroplast DNA can be used for the identification of plants as a barcode [26,27].

Molecular phylogenetic tools were broadly used to assess previous taxonomy and evolutionary processes. The number of chromosomes and contents of DNA (cytogenetic data) are usually associated with phylogenetic trees in order to understand the karyological differences involved in the group diversification [28,29]. Molecular phylogenomic studies using cp genome sequence data from the genes and slowly evolving inverted repeat regions have been applied to reveal the deep-level evolutionary relationships of plant taxa [30], producing robust phylogenies that are corroborated by sequence data from mitochondrial and nuclear genomes [31]. With the advancement of different genomic methods and tools, next-generation technologies have provided the rapid sequencing of different cp genomes from both angiosperms and gymnosperms in recent years, which enabled the confirmation of evolutionary relationships and detailed phylogenetic classifications to be conducted at the group, family, genus, and even species levels in plants [32,33]. Therefore, cp genome-scale data have increasingly been used to infer phylogenetic relationships at high taxonomic levels, and even at lower levels, great progress has been made [27,34,35,36].

The phylogenetic analysis of various *Oxalis* taxa has been reported previously by using nuclear Internal Transcribed Spacer (ITS) regions [37]; however, more detailed insight is still missing, keeping in view the complex taxonomy of *O. corniculata*. It is variable cytologically and genetically, but it is also phenotypically plastic [38,39]. Its taxonomy is complicated by the description of many subspecific taxa and other species now considered to be synonyms [5]. Furthermore, there has been taxonomic confusion of *O. corniculata* with closely related species, such as *O. stricta* and *O. dilleniid* [4]. Similarly, there is little information available on their genetic structure, especially their chloroplast genomes or their detailed phylogenetic placement. Hence, the current study was performed with the aim to sequence and analyze the complete chloroplast genome of *O. corniculata* and compare it with related species from the family Oxilidaceae (*Oxalis drummondii*, *Averrhoa carambola* and *Cephalotus follicularis*). We also aimed to elucidate and compare the global pattern of structural variation in the cp genome of *O. corniculata* and related three species. In addition, we compared the IR region contraction and expansion, intron contents, regions of high sequence divergence, and phylogenomic of *O. corniculata* with related species cp genomes to reveal more insight regarding the comparative genome architecture.

## 2. Results

### 2.1. Chloroplast Genome Structure of O. corniculata

**Figure 1:**
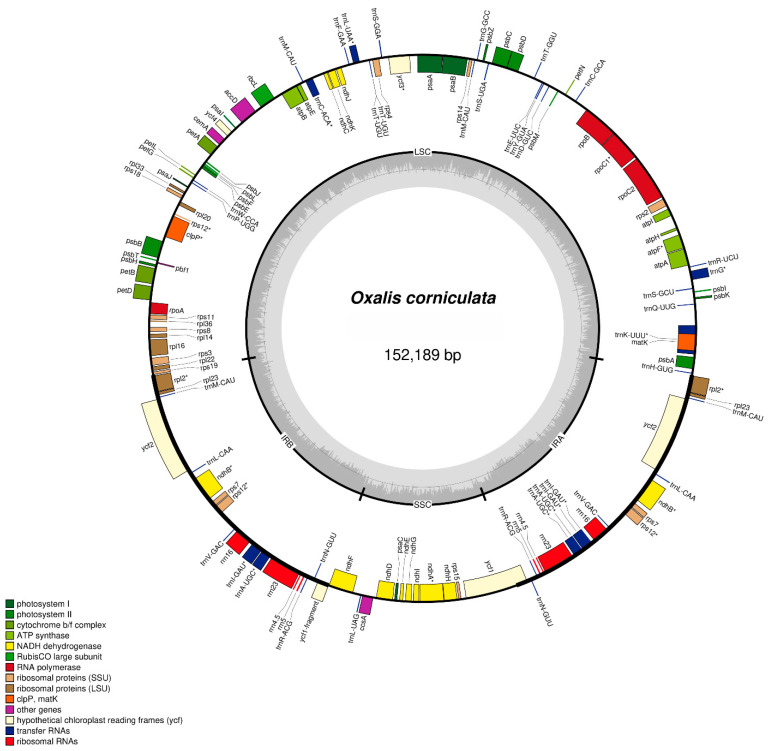
Gene map of the *O. corniculata* cp genome. Thick lines in the red area indicate the extent of the inverted repeat regions (IRa and IRb; 25,387 bp), which separate the genome into small (SSC; 16,990 bp) and large (LSC; 83,427 bp) single copy regions. Genes drawn inside the circle are transcribed clockwise, and those outsides are transcribed counter clockwise. Genes belonging to different functional groups are color-coded. The dark gray in the inner circle corresponds to the GC content, and the light gray corresponds to the AT content.

The chloroplast genome of *O. corniculata* is 152,189 bp and displays a distinctive quadripartite structure with a pair of 25,387 bp inverted repeats (IRs) that separate 83,427 bp single copy regions and 16,990 bp single copy regions (Figure 1). The total GC content is 36.7% with uneven distribution across the whole genome. The GC contents of IRs are higher (42.6%) than the large single copy and small single copy regions (34.4% and 30.3%, respectively). Furthermore, the *O. corniculata* cp genome consists of 131 genes and among all these genes, 82 are protein-coding genes, 40 are tRNA, and 8 are rRNA (Figure 1; Table 1). The protein-coding genes present in the *O. corniculata* cp genome included nine genes for large ribosomal proteins (*rpl*2, *14*, *16*, *20*, *22*, *23*, *32*, *33*, *36*), 11 genes for small ribosomal proteins (*rps*2, 3, 4, 7, 8, 11, 12, 14, 15, 18, 19), five genes for photosystem I (*psa*A, B, C, I, J), 15 genes for photosystem II (*psb*A, B, C, D, E, F, H, I, J, K, L, M, T, Z) and six genes (*atp*A, B, E, F, H, I) for ATP synthesis and the electron transport chain (Figure 1, Table 2). Similarly, 17 genes contain introns (11 protein-coding genes and 6 tRNA genes), of which three comprised of two introns (*rps*12, *clpP*, *ycf*3), while the rest have a single intron (Table 3). The small ribosomal protein 12 gene (rps12) is trans-spliced with a single intron. Its 5’ exon is located in the LSC region, while is 3’ exon is located in the IRb region and duplicated in the IRa region (Figure 1). The largest intron was present in *trn*K-UUU (2558 bp), whilst *trn*L-UAA contains the smallest intron (492 bp) (Table 3). The protein-coding region accounts for 52%, while the tRNA and rRNA regions constitute 1.99% and 5.94%, respectively, in the cp genome. The length of the protein-coding region is about 79,239 bp while those of tRNA and rRNA are 3042 bp and 9048 bp, respectively. Similarly, the *rps*16 gene, which is found in most angiosperm plastid genomes, is absent in *O. corniculata*. Furthermore, *inf*A is also absent in the *O. corniculata* cp genome.

### 2.2. Comparative Analysis of O. corniculata Chloroplast Genome with Related Species

**Figure 2:**
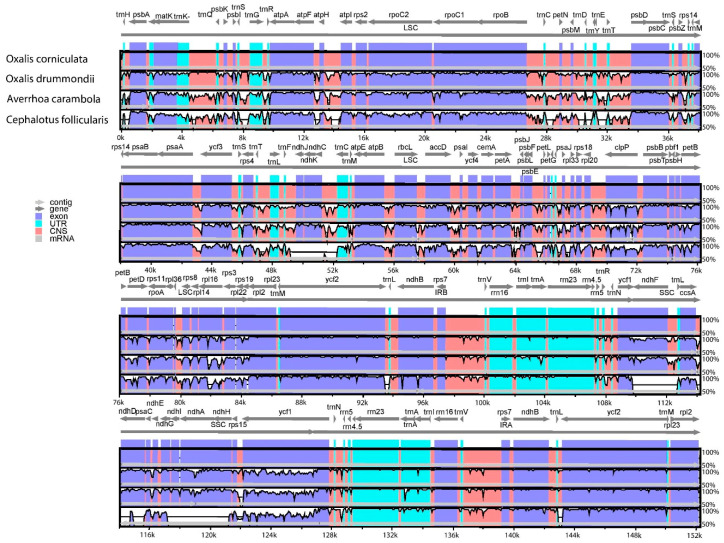
Visual alignment of plastid genomes of *O. corniculata* and three other members (*O. drummondii*, *A. carambola*, and *C. follicularis*) from the family Oxalidaceae. VISTA-based identity plot showing sequence identity among three species, using *O. corniculata* as a reference. The vertical scale indicates percent identity, ranging from 50% to 100%. The horizontal axis indicates the coordinates within the chloroplast genome. Arrows indicate the annotated genes and their transcription direction. The thick black lines show the inverted repeats (IRs).

**Figure 3:**
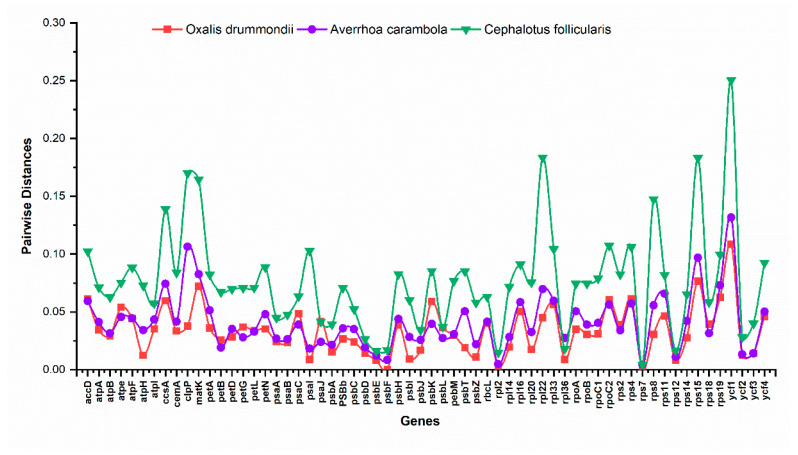
Pairwise sequence distance of 63 genes from of *O. corniculata* and three related species (*O. drummondii*, *A. carambola*, and *C. follicularis*) from family Oxidalaceae.

The *O. corniculata* chloroplast genome was compared with three already sequenced genomes from family Oxalidaceae i.e., *O. drummondii*, *A. carambola*, and *C. follicularis* (Table 1). Variations were observed in cp genomes where *C. follicularis* has the smallest cp genome, 142,706 bp, whilst the *A. carambola* cp genome was the largest, 155,965 bp, amongst analyzed species. We also compared the *O. corniculata* cp genome for pairwise sequence divergence (Table S1). Results showed that *O. corniculata* exhibited the lowest pairwise sequence divergence as compared to *O. drummondii* (0.044) and *A. carambola* (0.057; Table S1). Similarly, the whole cp genomes of *O. corniculata* were compared to identify sequence divergence via mVISTA (Figure 2). Results showed that the coding regions of all cp genomes are conserved compare to non-coding regions, whilst non-coding regions showed a higher divergence rate than the coding regions. The most divergence was observed in intergenic spaces. The *mat*K gene exhibited a high degree of divergence in all genomes, but it was higher in *A. carambola* and *C. follicularis* as compared to others. Similarly, *rpo*C1, *rpo*C2, and *rpo*B exhibited more divergence in four cp genomes with the highest in *C. follicularis*. The region between *rpo*B and *psb*D genes showed a high degree of divergence in all species in the LSC. The *ndh*K and *rpl*16 genes showed significant divergence compared to the *ycf*2 gene with lesser values. The *ndh*B is less divergent in all species except *C. follicularis*, where it showed high divergence. Similarly, the *ndh*F gene is highly divergent in all cp genomes, while it is absent in the *C. follicularis* cp genome. In the SSC, the region between *ndh*G and *ndh*H is highly divergent and the cp genome of *C. follicularis* lack most of the NadH oxidoreductase genes. Furthermore, about 63 protein-coding gene sequences were compared to obtain the average pairwise sequence distance among these species. The results showed that a majority of the genes maintained low levels of average sequence divergence. A relatively lower sequence identity was observed between the chloroplast genomes of *O. corniculata* with related species, especially in the *ccsA*, *clpP*, *rps8*, *rps15*, *rpl22*, *matK*, and *ycf1* genes (Figure 3).

### 2.3. Expansion and Contraction of IR Regions

**Figure 4:**
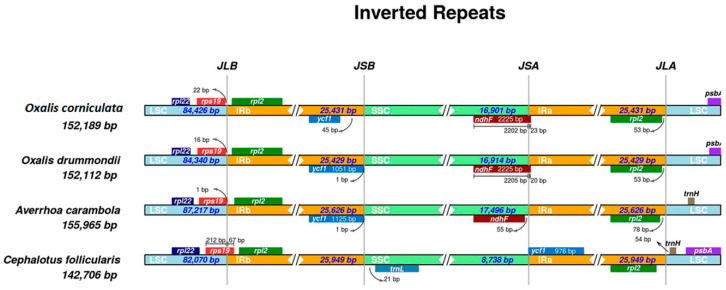
Distance between adjacent genes and junctions of the small single-copy (SSC), large single-copy (LSC), and two inverted repeat (IR) regions of *O. corniculata* with related species cp genomes. Boxes above and below the main line indicate the adjacent border genes. The figure is not to scale regarding sequence length, and it only shows relative changes at or near the IR/SC borders.

A detailed comparison was performed of the four junctions (J^LA^, J^LB^, J^SA^, and J^SB^) amongst IRs (IRa and IRb) and the LSC/SSC regions for the *O. corniculata*, *O. drummondii*, *A. carambola*, and *C. follicularis* (Figure 4). We carefully analyzed and compared the exact IR border positions and the adjacent genes among these cp genomes. The results revealed that at the LSC/IRb (JLB) border *rps19* gene is present 22 bp away from the JLB junction and located in the LSC region in *O. corniculata*. However, in *O. drummondii*, the *rps19* gene is located 16 bp away, while in *A. carambola*, it is 1 bp away from the JLB junction. On the other hand, in *C. follicularis*, *rps19* is located in the J^LB^ junction and extended 67 bp in the IRb region. The *ycf*1 gene is located 45 bp away from IRb/SSC (J^SB^) in *O. corniculata*, while in *O. drummondii* and *A. carambola*, the *ycf*1 gene is located 1 bp away in the IRb region. However, in *C. follicularis*, the *ycf*1 gene is absent in the IRb region and located 976 bp apart from the J^SA^ junction and located in the IRa region. Similarly, the *ndh*F gene is located in the (JSA) border and extended 23 bp and 20 bp in the IRa region in both *O. corniculata* and *O. drummondii* genomes, respectively, revealing the expansion of the IR region. In *A. carambola*, the *ndh*F gene is located 55 bp away from the J^SA^ junction in the SSC region. However, the *ndh*F gene is completely absent in the *C. follicularis* cp genome. Similarly, the *rpl2* gene is located 53 bp away from the IRa/LSC (J^LA^) junction in both *O. corniculata* and *O. drummondii*, while in *A. carambola* and *C. follicularis*, it is 78 bp and 124 bp away from the J^LA^ junction, respectively (Figure 4).

### 2.4. Repeat Sequence Analysis

**Figure 5:**
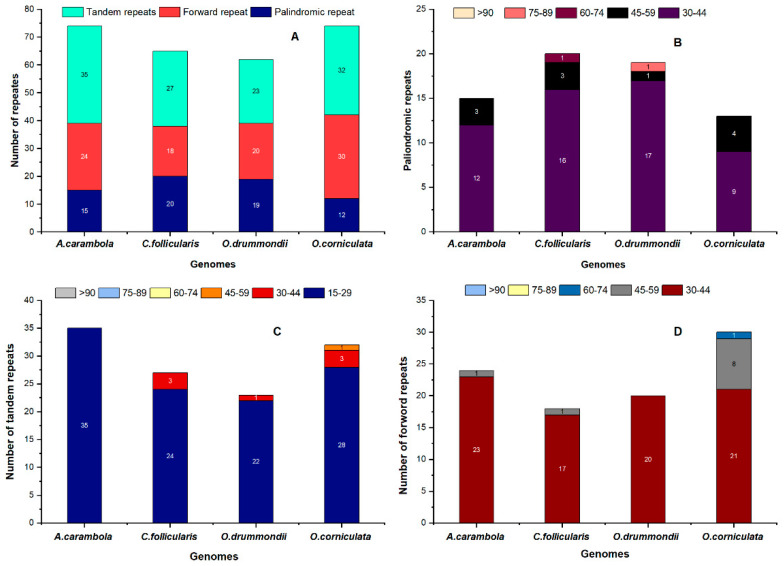
Analysis and graphical representation of repeated sequences in the four Oxalidaceae cp genomes. (**A**) Totals numbers of three repeat types; (**B**) Number of palindromic repeats by length; (**C**) Number of tandem repeats by length; (**D**) Number of forward repeats by length.

We investigated the repeat sequences of the *O. corniculata* chloroplast genome with the related species. The results revealed that *O. corniculata* contains 12 palindromic, 30 forward, and 32 tandem repeats, *O. drummondii* contains 19 palindromic, 20 forward, and 23 tandem repeats, *A. carambola* contains 15 palindromic, 18 forward, and 27 tandem repeats, and *C. follicularis* contain s20 palindromic, 30 forward, and 32 tandem repeats (Figure 5A). In *O. corniculata*, out of these repeats, the sizes of 9 palindromic repeats were 30–44 bp, while the sizes of 4 repeats were 45–59 bp. Likewise, the size of 28 and 3 tandem repeats were 15–29 bp and 30–44 bp, respectively, whereas the size of 21 forward repeats was found to be about 30–44 bp (Figure 5B–D). Amongst all these cp genomes 74 repeats (the highest) were detected in both *A. carambola* and *O. corniculata*. In all types of repeats, tandem repeats are the highest in number in all the cp genomes, followed by forward and palindromic repeats.

### 2.5. Simple Sequence Repeat (SSR) Analysis

**Figure 6:**
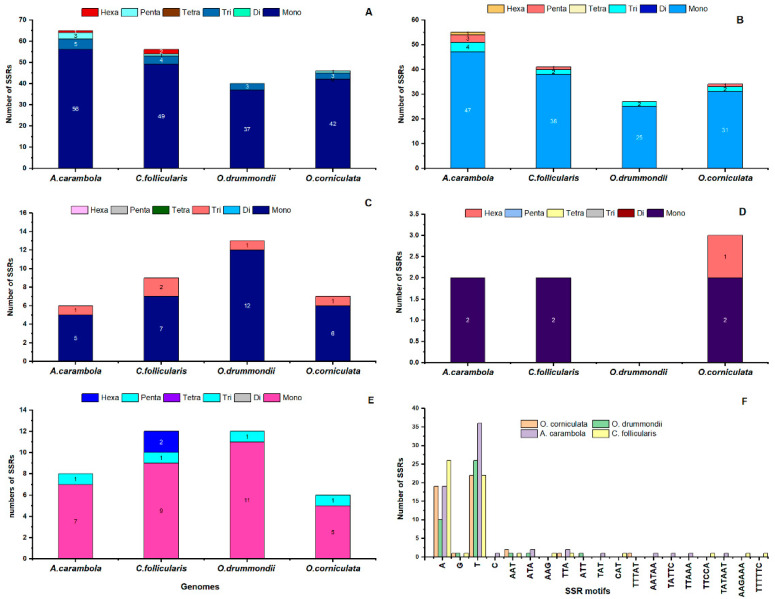
Analysis and graphical representation of simple sequence repeats (SSRs). Analysis of simple sequence repeats in the four Oxalidaceae chloroplast genomes (**A**), SSR numbers detected in the four species’ LSC regions (**B**), SSR numbers detected in the four species’ SSC regions (**C**), SSR numbers detected in the four species’ IR regions (**D**), Frequency of identified SSRs in the coding sequences (CDS) region (**E**) and frequency of identified SSR motifs in different repeat class types (**F**).

In simple sequence repeats (SSRs) analyses, a total of 46 SSRs were detected in the *O. corniculata* genome; among them, 42 are mononucleotide repeats, 3 are trinucleotide repeats, and 1 is a pentanucleotides repeat (Figure 6). There are no dinucleotides, tetranucleotides, and hexanucleotides in the *O. corniculata* genome. In *O. corniculata*, 13% SSRs are present in the CDS region, 73.9% is present in the LSC region, 15.2% is present in the SSC region, and 2.1% is present in the IR region (Figure 6B–E). Similarly, the highest numbers of SSRs in the other three species are located in the intergenic regions; i.e., *O. drummondii* (70%), *A. carambola* (87.6%), and *C. follicularis* (78.5%) followed by the LSC region—that is, 67.5%, 84.6%, and 73.2%, respectively (Figure 6B–E). On the other hand, in the cp genome of *O. drummondii*, a total of 40 SSRs were found, of which 37 are mono and 3 are trinucleotide, while di, tetra, Penta and hexanucleotides were not detected. In *A. carambola* and *C. follicularis*, 56 and 49 are mononucleotide repeats (Figure 6F). *A. carambola* have 5 trinucleotides, 3 penta, and 1 hexanucleotides repeat, while di and tetranucleotides repeats were missing. Similarly, in *C. follicularis*, 4 tri, 1 penta, and 2 hexanucleotides repeats were found, while di and tetranucleotides repeats were absent in this cp genome. Among the four cp genomes, *A. carambola* has a high number of SSRs; i.e., 56.

### 2.6. Phylogenetic Analysis

**Figure 7:**
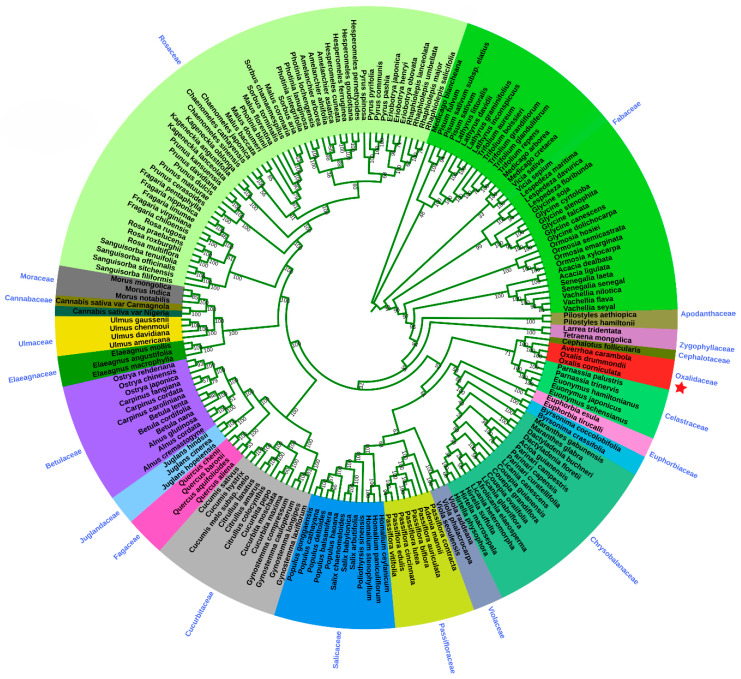
Phylogenetic trees of *O. corniculata* with related species. The 60 shared genes dataset was analyzed by using maximum likelihood (ML). Numbers above the branches represent bootstrap values in the ML. The red star represents the position of *O. corniculata* (MN998500).

For the phylogenetic analysis of *O. corniculata*, we have downloaded about 191 genomes from the 20 families mentioned in the Materials and Methods section. We inferred the phylogenetic position of *O. corniculata* on the basis of 60 shared genes among these genomes. The study revealed that *O. corniculata* forms a single clade with *O. drummondii* and *A. carambola* in the family Oxalidaceae (Figure 7). These results also showed that *O. corniculata* is closer to *O. drummondii* than *A. carambola*, which is a different genus. Furthermore, the phylogenetic tree also inferred that the Oxalidaceae family is close to Cephalotaceae and Celastraceae with high bootstrap support (100%), followed by Zygophyllaceae and Euphorbiaceae. The phylogenetic trees in this study also exhibited that Rosaceae is highly interlinked to Moraceae. Similarly, the phylogenetic trees also indicate the close relationship of Fabaceae with Apodanthaceae.

## 3. Discussion

In case of genetic and evolutionary relationship assessments among plant species, chloroplast DNA sequences have been extensively used [40,41,42]. The complete chloroplast genome sequences provided sufficient information to reconstruct both current and prehistoric diversifications [43]. The powerful and flexible nature of Next Generation Sequencing (NGS) has permeated many areas of study, enabling the development of a broad range of applications that have transformed study designs capable of unlocking information of the genome, transcriptome, and epigenome of any organism [44]. In the current study, we have sequenced the complete genome of *O. corniculata* chloroplast for the first time. The results revealed that the chloroplast genome size of *O. corniculata* is in line with the chloroplast of those flowering plants, which ranges from 125,373 bp to 176,045 bp in *Cuscuta exaltata* and *Vaccinium macrocarpon*, respectively [45,46]. The CG content of *O. corniculata* is 36.7% (Table 1), which is slightly lower than *C. follicularis* and *Paeonia obovata* (38.43%) [47]. The GC content in the IR region is higher (42.6%) than that of the LSC and SSC regions. As a result of the presence of the rich GC nucleotide, higher GC content was present in the IR region of rRNA genes such as *rrn*5, *rrn*4.5, *rrn*23, and *rrn*16, which is consistent with what has been investigated in other cp genomes [48,49,50].

In most angiosperms, it is believed that the gene(s) of the chloroplast genome and their organization are extremely conserved [51]. In correlation, we detected 131 genes in the cp genome of *O. corniculata* while other studies also show that many angiosperms have retained these genes [52,53]. With the increasing number of chloroplast genome sequences, the diverse organization of the chloroplast genome is becoming more evident, as demonstrated by genome rearrangement and gene losses in the chloroplast genomes of Oxiladaceae. For example, the *rps16* gene, which is found in most angiosperm plastid genomes, has been lost in *O. corniculata*. Similar results have been reported in various cp genomes previously [54]. Furthermore, in *O. corniculata*, the cp genome the *infA* gene was lost, as reported previously by various researchers, the *infA* gene has been independently lost multiple times from angiosperms and especially in most Rosids [32,51]. Moreover, we found 11 protein-coding genes and 6 tRNAs genes containing introns in the *O. corniculata* cp genome. Among them, three genes, *clp*P, *rps12*, and *ycf*3 have two introns, while the others have one intron. Similar results were also reported previously in the *Manihot esculenta* chloroplast genome [32] and *Oresitrophe* chloroplast genome [55]. In this study, genes *ccsA, clpP, rps8, rps15, rpl22, matK*, and *ycf1* were found to have high evolution rates among the four cp genomes (Figure 3), which agreed with earlier reports of Cuenoud et al. [41]. Similar results of these genes were reported previously among 17 vascular plants and *Panax* species [56].

In the terrestrial plants, the cp genome is very conserved structurally, and the large inverted repeats (IRs) junction is not essential to the function of the cp genome [57]. It is believed that IRs are the most conserved region due to which the rate of natural nucleotide substitution in IRs is lesser as compared to single copy regions, and the variation in IR/LSC and IR/SSC boundaries is the key reason for the size variation among the cp genomes of different groups. The variation in size among four genomes was exhibited by the slight expansion of the IRb (JLB border) in *C. follicularis* compared to *O. corniculata* (Figure 4). These results are in agreement with previous work where IRs are one of the efficient tools for conformational reorganizations within the plastids genomes and are regularly subjected to expansion, contraction, or even complete loss [20]. Similarly, previous results showed that contractions and expansions of the IR regions triggered the diversification of size among the cp genomes [58].

The study of different repeats (palindromic, forward, and tandem) in our sequenced cp genome showed variation in the number of repeats, which is similar to other species previously studied [59]. In all types of repeats, tandem repeats were found more than palindromic and forward repeats in four cp genomes; these results are consistent with previous reports of Teucrium and Commiphora species [60,61], as well as *S. miltiorrhiza* [62]. Similarly, simple sequence repeats (SSRs) usually have a higher rate of mutation compared with other neutral regions of DNA due to slipped strand mispairing. In genetic studies, due to the haploid and nonparental inheritance nature of cp SSRs, they are commonly used for the assessment of population structure as molecular markers [63,64]. In this study, we comparatively studied the ideal SSRs among the four species *O. corniculata*, *O. drummondii*, *A. carambola*, and *C. follicularis* (Figure 6). The largest number of SSRs was found in *A. carambola*, followed by *C. follicularis*. Mononucleotide repeats were found to be the most common type of SSR in all four species; the A or T mononucleotide repeats are most abundant SSRs in *O. corniculata* (Figure 6), which is congruent to the previous result that SSRs in the chloroplast genome are commonly composed of A or T repeats and rarely G or C repeats [62,65].

Recently, cp genomes information has provided a large amount of data for improving phylogenetic resolution. Chloroplast genome sequences have been widely used for the reconstruction of phylogenetic relationships among plant lineages [66,67,68,69]. The phylogenetic evaluation of plant species might not be easy to resolve evolutionary relationships, specifically at taxonomic levels while using a small number of loci [70,71]. Previous phylogenetic studies based on the complete cp genomes and shared genes have been used to explain problematic phylogenetic relations among nearly associated species [34,68] and to increase our concept related to evolutionary relations of angiosperms [72,73]. Phylogenetic relationships of *O. corniculata* were inferred by using 60 shared genes datasets by using the ML method. The results showed that *O. cornicualata* form a single clade with *O. drummondii*. Similarly, the phylogenetic tree also inferred that Oxalidaceae family is close to Cephalotaceae and Celastraceae with high bootstrap support (100%), followed by Zygophyllaceae and Euphorbiaceae (Figure 7). Further cp genomes from the family Oxalidaceae should be explored to determine the phylogenetic position of *O. corniculata* within the section Corniculatae.

## 4. Materials and Methods

### 4.1. Chloroplast DNA Extraction, Sequencing, and Assembly

Young and immature leaves of *O. corniculata* were ground into fine powder in liquid nitrogen, and pure DNA was isolated through a DNeasy Plant Mini Kit (Qiagen, Valencia, CA, USA). The resultant chloroplast DNA, by using an Illumina HiSeq-2000 platform (San Diego, CA, USA) at Macrogen (Seoul, Korea) was sequenced. A total of 43,453,336 raw reads were generated for *O. corniculata*, and CLC Genomics Workbench v7.0 (CLC Bio, Aarhus, Denmark) was used to trim and filter reads for the de novo genome assembly. Trimmomatic 0.36 was used for filtering the reads and trailing and leading nucleotide with a Phred score of <20 or when the Phred score dropped below 20 on implementing a 4-bp sliding-window approach. Similarly, reads of <50 bp were discarded after quality filtering and adaptor trimming. The first assembly was formed using SPADES v3.9.0, with an additional switchover to SOAP denovo v2.04. The resulting contigs were compared against the chloroplast genomes of *O. drummondii* using BLASTN with an E-value cut-off of 1 × 10^−5^. The uncertain regions in these genomes, such as IR junction’s region, were chosen from the already published genome mentioned above to adjust the sequence length using the iteration method and by employing the Geneious v11.1.2 software [74]. The chloroplast genome sequence of *O. corniculata* has been submitted to GenBank (accession number: MN998500).

### 4.2. Genome Annotation

The Dual Organellar Genome Annotator (DOGMA) [75] was used to annotate the cp genomes of the sequenced species and through BLASTX, the number and position of ribosomal RNAs, transfer RNAs, and other coding genes present in chloroplast genomes were identified and analyzed, while BLASTN tRNAscan-SE version 1.21 was used for tRNA annotation [76] software. Furthermore, Geneious (v11.0) and tRNAscan-SE [76] were used for manual adjustment to compare with the reference genomes reported previously. Similarly, the start and stop codon and intron boundaries were also manually adjusted and compared with the reference chloroplast genome already published. Additionally, by using Organellar Genome DRAW (OGDRAW) [77], the structural characteristics of chloroplast genomes of *O. corniculata* were demonstrated. Beside this, to determine the relative synonymous codon usage and deviations in synonymous codon usage by avoiding the effect of amino acid composition, MEGA6 software [78] was used.

### 4.3. Characterization of Repetitive Sequences and SSR

REPuter software [79] was used to determine the repetitive sequences (palindromic, reverse and direct repeats) within these four cp genomes (*O. corniculata*, *O. drummondii*, *A. carambola*, and *C. follicularis*). Subsequent settings were used for repeat identification through REPuter: (1) a minimum repeat size of 30 bp, (2) ≥90% sequence identity, and (3) a Hamming distance of 1. Tandem Repeats Finder version 4.07 b was used to find tandem repeats by using default settings [80]. The MIcroSAtellite (MISA) identification tool was used for the microsatellite analysis of *O. corniculata* and another three species’ (*O. drummondii*, *A. carambola*, and *C. follicularis*) cp genomes [81]. The parameters such as unit_size and min_repeats were defined as follows: 1–10, 2–8, 3–4, 4–4, 5–3, and 6–3; the smallest distance between two SSRs was set to 100 bp. The following conditions were set for parametric significance: 10 or more repeats of one base, 6 or more repeats of two bases, 5 or more repeats of three bases, 5 or more repeats of four bases, 4 or more repeats of five bases, and 4 or more repeats of six bases.

### 4.4. Sequence Divergence and Phylogenetic Analysis

In the *O. corniculata* chloroplast genome, the average pairwise sequence divergence with three related species (*O. drummondii*, *A. carambola*, and *C. follicularis*) from the family Oxalidaceae was determined. After a comparison of gene order and multiple sequence alignment, comparative sequence analysis was used to recognize missing and unclear gene annotations. For whole genome alignment, MAFFT version 7.222 [82], with default parameters were used, and pairwise sequence divergence was calculated by the use of the selected Kimura’s two-parameter (K2P) model [83]. MEGA 6 software [78] was used to evaluate the relative synonymous codon usage by avoiding the effect of amino acid composition. Finally, the divergence of the new *O. corniculata* cp genomes from related species of family Oxalidaceae was determined using mVISTA [84] in Shuffle-LAGAN mode and by employing the genome of new *O. corniculata* as a reference. To resolve the phylogenetic position of *O. corniculata* within the family Oxalidaceae and to check the relationship of 20 families (Fabaceae, Apodanthaceae, Zygophyllaceae, Cephalotaceae, Oxalidaceae, Celastraceae, Euphorbiaceae, Malpighiaceae, Chrysobalanaceae, Violaceae, Passifloraceae, Salicaceae, Cucurbitaceae, Fagaceae, Juglandaceae, Betulaceae, Elaeagnaceae, Ulmaceae, Cannabaceae, Moraceae, Rosaceae) in monophyletic clade rosids, about 60 share genes from 191 cp genomes were downloaded from the National Center for Biotechnology Information (NCBI) database. For the alignment of 60 shared genes, MAFFT version 7.222 [82] with default parameters was used. The maximum likelihood (ML) method was adopted to infer the phylogenetic trees with MEGA 6 [78], and parameters were adjusted with a BIONJ tree with 1000 bootstrap replicates using the Kimura two-parameter model with gamma-distributed rate heterogeneity and invariant sites.

## 5. Conclusions

The current findings reveal detailed understandings of the complete cp genome of *O. corniculata* for the first time through sequencing on Illumina HiSeq-2000 platform. The gene order and gene structure of *O. corniculata* was found to be similar with three related species from the family Oxalidaceae. Through detailed bioinformatic analysis and comparative assessments, we retrieved essential genetic features such as repetitive sequences, SSRs, codon usage, IR contraction and expansion, sequence divergence, and phylogenomic placement. Whole cp genome comparisons revealed an overall high degree of sequence similarity between *O. corniculata* and *O. drummondii* and some divergence in the intergenic spacers of other species. No major structural rearrangement in these four cp genomes was observed. Phylogenomic analyses of the complete plastid genomes revealed that *O. corniculata* is closely related to *O. drummondii*. A current plastome genomic dataset and the detailed analysis of *O. corniculata* and related species and their comparative analysis provide a powerful genetic resource for the future molecular phylogeny, evolution, population genetics, and biological functions of genus *Oxalis*.

## Figures and Tables

**Table 1:** Summary of complete chloroplast genome of *O*. *corniculata* and its comparison with related species from family Oxalidaceae.

	*A. carambola*	*C. follicularis*	*O. drummondii*	*O. corniculata*
Size (bp)	155,965	142,706	152,112	152,189
Overall GC contents	36.5	37.1	36.5	36.7
LSC size in bp	86,218	81,071	83,341	83,427
SSC size in bp	17,497	8739	16915	16,990
IR size in bp	25,626	25,949	25,429	25,387
Protein coding regions size in bp	78,528	66,672	77,826	79,239
tRNA size in bp	2790	2790	2790	3042
rRNA size in bp	9046	9050	9046	9048
Number of genes	131	122	127	131
Number of protein coding genes	83	71	82	83
Number of rRNA	8	8	8	8
Number of tRNA	37	37	37	40

**Table 2:** Genes in the sequenced *O. corniculata* chloroplast genome.

Category	Group of Genes	Name of Genes
Self-replication	Large subunit of ribosomal proteins	*rpl*2 *, *rpl*14, *rpl*16, *rpl*20, *rpl*22, *rpl*23 *, *rpl*33, *rpl*36
	Small subunit of ribosomal proteins	*rps*2, *rps*3, *rps*4, *rps*7 *, *rps*8, *rps*11, *rps*12 *, *rps*14, *rps*15, *rps*18, *rps*19
	DNA dependent RNA polymerase	*rpo*A, *rpo*B, *rpo*C1, *rpo*C2
	rRNA genes	*rrn*4.5, 5, 16, 23
	tRNA genes	*trn*A-UGC *, *trn*C-GCA, *trn*D-GUC, *trn*E-UUC , *trn*F-GAA, *trn*fM-CAU, *trn*G-GCC *, *trn*H-GUG, *trn*I-GAU *, *trn*K-UUU, *trn*L-CAA *, *trn*L-UAA, *trn*L-UAG, *trn*M-CAU *, *trn*N-GUU *, *trn*P-UGG, *trn*Q-UUG, *trn*R-ACG *, *trn*R-UCU, *trn*S-GCU, *trn*S-GGA, *trn*S-UGA, *trn*T-GGU, *trn*T-UGU *, *trn*V-GAC *, *trn*W-CCA, *trn*Y-GUA
Photosynthesis	Photosystem I	*psa*A, B, C, I, J
	Photosystem II	*psb*A, *psb*B, *psb*C, *psb*D, *psb*E, *psb*F, *psb*H, *psb*I, *psb*J, *psb*K, *psb*L, *psb*M, *psb*T, *psb*Z
	NadH oxidoreductase	*ndh*A, *ndh*B *, *ndh*C, *ndh*D, *ndh*E, *ndh*F, *ndh*G, *ndh*H, *ndh*I, *ndh*J, *ndh*K
	Cytochrome b6/f complex	*pet*A, *pet*B, *pet*D, *pet* G, *pet*L, *pet*N
	ATP synthase	*atp*A, *atp*B, *atp*E, *atp*F, *atp*H, *atp*I
	Rubisco	*rbcL*
Other genes	Maturase	*mat*K
	Protease	*clp*P
	Envelop membrane protein	*cem*A
	Subunit Acetyl- CoA-Carboxylate	*acc*D
	c-type cytochrome synthesis gene	*ccs*A
Unknown	Conserved Open reading frames	*ycf1* *, *2* *, *3*, *4*,

* Duplicated genes.

**Table 3:** The genes with introns in the four species chloroplast genome and the length of exons and introns.

Gene	Location	Exon I (bp)				Intron 1 (bp)				Exon II (bp)				Intron II (bp)				Exon III (bp)			
		*O. c*	*O. d*	*A. c*	*C. f*	*O. c*	*O. d*	*A. c*	*C. f*	*O. c*	*O. d*	*A. c*	*C. f*	*O. c*	*O. d*	*A. c*	*C. f*	*O. c*	*O. d*	*A. c*	*C. f*
*atpF*	LSC	144	145	145	145	717	714	714	717	411	410	410	410								
*petB*	LSC	6	6	6	6	746	746	785	793	645	642	642	642								
*PetD*	LSC	8	8	7	7	746	778	707	721	645	475	473	473								
*rpl2* *	IR	391	391	391	391	658	661	659	671	434	434	434	525								
*rps16*	LSC	--	--	40	40	--	--	935	929	--	--	224	227								
*rpoC1*	LSC	430	432	453	459	727	729	751	759	1634	1629	1608	1617								
*rps12* *	IR/LSC			391		527				232		434						25			
*clpP*	LSC	71	71	69	69	808	812	833	833	289	289	291	291		603	612	568		228	228	228
*ndhA*	SSC	557	557	559	--	1077	1067	1037	--	541	541	557	--								
ndhB*	IR	777	777	777	--	685	685	685	--	756	756	756	--								
*ycf3*	LSC	124	124	126	126	718	712	718	714	228	230	228	228	684	678	685	678	155	153	153	150
*trnA-UGC* *	IR	38	38	38	38	799	798	763	811	35	35	35	35								
*trnI –GAU* *	IR	37	37	37	37	925	924	932	946	35	35	35	35								
*trnL-UAA*	LSC	35	35	35	35	497	497	507	492	50	50	50	50								
*trnK -UUU*	LSC	37	34	37	35	2519	2515	2545	2558	35	37	35	37								
*trnG-GCC*	LSC	71	71	71	71	--	--	--	--	--	--	--	--								
*trnV-UAC*	LSC	--	--	35	35	--	--	617	618	--	--	39	39								

O. c = *Oxalis corniculata*, O. d = *Oxalis drummondii*, A. c = *Averrhoa carambola*, C. f = *Cephalotus follicularis*.

## Data Availability

All data generated or analyzed during this study are included in this published article.

## Supplementary Materials

The following are available online at https://www.mdpi.com/2223-7747/9/8/928/s1, Table S1. Average pairwise sequence distance of *O. corniculata* with related species cp genomes.
